# Maternal N-Acetylcysteine Therapy Prevents Hypertension in Spontaneously Hypertensive Rat Offspring: Implications of Hydrogen Sulfide-Generating Pathway and Gut Microbiota

**DOI:** 10.3390/antiox9090856

**Published:** 2020-09-13

**Authors:** Chien-Ning Hsu, Chih-Yao Hou, Guo-Ping Chang-Chien, Sufan Lin, You-Lin Tain

**Affiliations:** 1 Department of Pharmacy, Kaohsiung Chang Gung Memorial Hospital, Kaohsiung 833, Taiwan; chien_ning_hsu@hotmail.com; 2 School of Pharmacy, Kaohsiung Medical University, Kaohsiung 807, Taiwan; 3 Department of Seafood Science, National Kaohsiung University of Science and Technology, Kaohsiung 811, Taiwan; chihyaohou@webmail.nkmu.edu.tw; 4 Center for Environmental Toxin and Emerging-Contaminant Research, Cheng Shiu University, Kaohsiung 833, Taiwan; guoping@csu.edu.tw (G.-P.C.-C.); linsufan2003@gmail.com (S.L.); 5 Super Micro Mass Research and Technology Center, Cheng Shiu University, Kaohsiung 833, Taiwan; 6 Department of Pediatrics, Kaohsiung Chang Gung Memorial Hospital and Chang Gung University College of Medicine, Kaohsiung 833, Taiwan; 7 Institute for Translational Research in Biomedicine, Kaohsiung Chang Gung Memorial Hospital and Chang Gung University College of Medicine, Kaohsiung 833, Taiwan

**Keywords:** developmental origins of health and disease (DOHaD), gut microbiota, hydrogen sulfide, hypertension, N-acetylcysteine, oxidative stress, renin-angiotensin system, thiosulfate

## Abstract

Hypertension can come from early life. N-acetylcysteine (NAC), a hydrogen sulfide (H_2_S) precursor as well as an antioxidant, has antihypertensive effect. We investigated whether maternal NAC therapy can protect spontaneously hypertensive rats (SHR) male offspring against hypertension. The pregnant rats were assigned to four groups: SHRs without treatment; Wistar Kyoto (WKY) without treatment; SHR+NAC, SHRs received 1% NAC in drinking water throughout pregnancy and lactation; and, WKY+NAC, WKY rats received 1% NAC in drinking water during pregnancy and lactation. Male offspring (*n* = 8/group) were killed at 12 weeks of age. Maternal NAC therapy prevented the rise in systolic blood pressure (BP) in male SHR offspring at 12 weeks of age. Renal cystathionine β-synthase (CBS) and 3-mercaptopyruvate sulphurtransferase (3MST) protein levels and H_2_S-releasing activity were increased in the SHR+NAC offspring. Maternal NAC therapy increased fecal H_2_S and thiosulfate levels in the SHR+NAC group. Additionally, maternal NAC therapy differentially shaped gut microbiota and caused a distinct enterotype in each group. The protective effect of maternal NAC therapy against hypertension in SHR offspring is related to increased phylum *Actinobacteria* and genera *Bifidobacterium* and *Allobaculum*, but decreased phylum *Verrucomicrobia*, genera *Turicibacter*, and *Akkermansia*. Several microbes were identified as microbial markers, including genera *Bifidobacterium, Allobaculum, Holdemania*, and *Turicibacter*. Our results indicated that antioxidant therapy by NAC in pregnant SHRs can prevent the developmental programming of hypertension in male adult offspring. Our findings highlight the interrelationships among H_2_S-generating pathway in the kidneys and gut, gut microbiota, and hypertension. The implications of maternal NAC therapy elicited long-term protective effects on hypertension in later life that still await further clinical translation.

## 1. Introduction

Hypertension is a common disease and a leading global risk for mortality [1]. Hypertension can originate in early life by so-called “developmental origins of health and disease” (DOHaD) [2,3]. Accumulative evidence demonstrates that early-life oxidative stress can increase the risk of developing hypertension in later life [3,4,5]. Conversely, antioxidants have shown benefits in hypertension by counteracting oxidative stress [6]. Blood pressure (BP) regulation is mainly governed by the kidneys. During development, the kidneys can be programmed by adverse early-life environments, namely renal programming, leading to hypertension in adulthood [7,8]. In the kidneys, the imbalance between vasodilators, such as nitric oxide (NO), and vasoconstrictors, such as angiotensin II (Ang II), in favor of vasoconstriction can lead to hypertension [9]. We and others have demonstrated that renal NO deficiency is characterized in the prehypertensive stage of the spontaneously hypertensive rats (SHRs), a commonly used genetic hypertensive model [10,11]. 

Like NO, hydrogen sulfide (H_2_S) has recently emerged as a vasodilator involved in hypertension [12]. In SHR, H_2_S deficiency appears prior to the development of hypertension, whereas the administration of exogenous H_2_S protects SHRs against hypertension [13,14]. All of the H_2_S-generating enzymes are expressed in the kidneys, including cystathionine γ-lyase (CSE), cystathionine β-synthase (CBS), and 3-mercaptopyruvate sulphurtransferase (3MST) [15]. L-cysteine is the major precursor for endogenous H_2_S synthesis [12]. Our previous study reported that early supplementation of L-cysteine in the prehypertensive stage can protect SHRs against high-salt-enhanced elevation of BP and kidney injury [16]. N-acetylcysteine (NAC), which is a stable analogue of L-cysteine, is currently mainly used as an antioxidant [17]. NAC has been shown a variety of health benefits, including the production of glutathione, antihypertensive effect, and H_2_S generation [17,18]. We previously observed that NAC therapy can prevent the development of hypertension in young SHR and in programmed hypertension models [19,20,21]. Nevertheless, whether maternal NAC supplementation can prevent adult SHR offspring against hypertension via the regulation of H_2_S-generating pathway in the kidneys and gut remains unclear.

Current evidence supports a pathogenic association between gut microbiota dysbiosis and hypertension via the gut-kidney axis [22]. Several possible mechanisms have been proposed that link the gut microbiota dysbiosis and hypertension, including alterations of gut microbiota compositions, dysregulation of the renin-angiotensin system (RAS), and inhibition of NO as well as H_2_S [23]. H_2_S can also be produced by bacteria residing within the gut. In the gut, sulfate-reducing (SRB) and sulfur-oxidizing bacteria (SOB) both contribute to the balance of the H_2_S level [24]. Approximately 50% of fecal H_2_S is derived from dissimilatory sulfate reduction by the SRB or assimilatory sulfate reduction by other bacteria [25]. H_2_S is considered to be a double-edge sword, with harmful effects at higher concentrations, but beneficial effect at low concentration [26]. Although SRB-derived H_2_S was reported to be deleterious in some diseases [27], the impact of SRB and SOB in hypertension is still largely unknown. Moreover, thiosulfate is a metabolite of H_2_S and also generates H_2_S through a reductive reaction [28]. In this manner, thiosulfate could serve as an advantageous recycling pathway to increase H_2_S bioavailability [29]. Therefore, we intended to analyze H_2_S and thiosulfate levels in the plasma and feces in order to determine the impact of gut bacteria-derived H_2_S on hypertension in this model. 

NAC therapy during pregnancy and lactation on the protection of offspring hypertension programmed by early-life insults have been reported in several animal models [19,20,30]. In the current study, we aimed to extend previous research by examining whether maternal NAC therapy can prevent hypertension in SHR offspring and whether its beneficial effects are relevant to mediating H_2_S-genetaing system in the gut and kidneys, the intrarenal RAS, and gut microbiota.

## 2. Materials and Methods 

### 2.1. Animals and Experimental Design

All of the animal experiments were performed in accordance with legislation on the Guide for the Care and Use of Laboratory Animals of the National Institutes of Health and were approved (Permit Number 2019050902; approval date: 4 November 2019) by the Institutional Animal Ethics Committee of Chang Gung Memorial Hospital. Virgin SHR and normotensive control Wistar Kyoto (WKY) rats purchased from BioLASCO Taiwan Co., Ltd. (Taipei, Taiwan) were used for breeding. The rats could acclimatize in a light/dark cycle of 12:12 h, temperature- (22 ± 1 °C), and humidity- (55 ± 5%) controlled room in a core animal facility accredited by the Association for Assessment and Accreditation of Laboratory Animal Care International. Male rats were caged with individual females until mating is confirmed by observation of a vaginal plug. Pregnant rats were assigned to four groups: Group1, SHR without treatment; Group 2, WKY without treatment; Group 3, SHR+NAC, rats receive 1% NAC in drinking water during three-week pregnancy and three-week lactation; and, Group 4, WKY+NAC, the rats receive 1% NAC in drinking water during tree-week pregnancy and three-week lactation. NAC dose used in this study is based on that used in our previous studies [19,20]. We only selected male offspring from each litter due to hypertension and kidney disease occurring at an earlier age and at a higher rate in males than females [31]. Male offspring were culled to eight pups after birth, in order to standardize the quantity of milk and maternal pup care received, and used in subsequent experiments. As we described previously [19], BP was measured in conscious rats every two weeks over the course of eight weeks while using an indirect tail-cuff method (BP-2000; Visitech Systems, Inc., Apex, NC, USA). All of the rats were acclimated to restraint and tail-cuff inflation for 1 week prior to the measurement. Every effort was made to ensure accuracy and reproducibility. All of the rats were killed at 12 weeks of age and stool samples were collected. Blood samples were collected in heparinized tubes. Kidneys were harvested after perfusion with phosphate buffered saline (PBS). Each kidney was decapsulated, washed with PBS, divided into the cortex and medulla regions, snap-frozen in liquid nitrogen, and stored at −80 °C. 

### 2.2. Quantitative Real-Time PCR Analysis

Two-step quantitative real-time PCR (qPCR) was conducted using Quantitect SYBR Green PCR Reagents (Qiagen, Valencia, CA) on an iCycler iQ Multi-color Real-Time PCR Detection System (Bio-Rad, Hercules, CA). We analyzed three H_2_S-generating enzymes, including CBS (*Cbs*), CSE (*Cth*), and 3MST (*Mpst*). Additionally, we determined the renal expression of several components in the RAS [32]. In the current study, *Ren* (renin), *atp6ap2* (prorenin receptor), *Ace1* (angiotensin converting enzyme-1), *Ace2*, *Agtr1* (angiotensin II type 1 receptor), *Agtr2* (angiotensin II type 2 receptor), and *Mas* (angiotensin (1–7) receptor MAS) were analyzed. We used *Rn18S* as a reference gene. Table 1 shows the primers used in this study. All of the samples were run in duplicate. We used the comparative threshold cycle (CT) method to determine the relative quantification of gene expression. The average C_T_ value for each sample was subtracted from each gene’s corresponding average *Rn18S* value to calculate the ΔC_T_. ΔΔC_T_ was calculated by subtracting the average control ΔC_T_ value from the average experimental ΔC_T_. We calculated the value of 2^−ΔΔCT^ to obtain the expression fold change.

### 2.3. Western Blot

The Western blot analysis was performed on the kidney cortex homogenate, as previously described [19]. We used 6–10% polyacrylamide gels and separated by electrophoresis (200 V, 90 min.), and then electrotransferred to a nitrocellulose membrane (GE Healthcare Bio-Sciences Corp., Piscataway, NJ, USA). Following transfer, the membrane was stained with Ponceau S red (PonS) stain solution (Sigma-Aldrich, St. Louis, MO, USA) for 10 min. on the rocker in order to verify equal loading. After blocking with phosphate-buffered saline-Tween (PBS-T) containing 5% dry milk and washing, the membrane was subsequently incubated with a primary antibody. We used the following antibodies: a mouse monoclonal anti-rat CBS antibody (1:1000, overnight incubation; Abnova Corporation, Taipei, Taiwan), a rabbit polyclonal anti-rat CSE antibody (1:1000, overnight incubation; Proteintech Group, Inc. Chicago, IL, USA), and a rabbit monoclonal anti-rat 3MST antibody (1:500, overnight incubation; Novus Biologicals, Littleton, CO, USA). The bands of interest were visualized while using a SuperSignal West Pico reagent (Pierce; Rockford, IL, USA). Densitometry analysis was provided for each band, and it was quantified as the integrated optical density (IOD) normalized to PonS staining. 

### 2.4. Renal H_2_S-Producing Capacity

According to a protocol that was validated in our lab [19], we analyzed H_2_S-producing capacity in kidneys [19]. In brief, kidney homogenates (430 μL) were incubated with pyridoxal 5-phosphate (2 mM; 20 μL), L-cysteine (10 mM; 20 μL), and 30 μL saline in the tightly sealed Eppendorf vials for 30 min. at 37 °C. Later, 250 μL 1% zinc acetate was injected. We added 250 μL 10% trichloroacetic acid to stop the reaction and precipitate proteins. Subsequently, N, N-dimethyl p-phenylenediamine sulfate (20 mM; 20 μL) in 7.2 M HCl was added followed by FeCl_3_ (30 mM; 20 μL) in 1.2 M HCl. After 30 min. at room temperature, the absorbance values were measured at 670 nm. All of the samples were run in duplicate, and concentration was calculated against a calibration curve of NaHS (3.125–250 μM). The tissue concentration was represented as nmol/gram protein/min., factored by the protein concentration.

### 2.5. High Performance Liquid Chromatography-Mass Spectrometry (HPLC-MS/MS) Analysis

We analyzed plasma and fecal concentrations of H_2_S and thiosulfate by high performance liquid chromatography-Mass Spectrometry (HPLC-MS/MS) analysis while using an Agilent Technologies 1290 HPLC system coupled with an Agilent 6470 Triple Quadrupole LC/MS (Agilent Technologies, Wilmington, DE, USA). Phenyl 4-hydroxybenzoate (PHB) was added to samples as an internal standard. The H_2_S derivative sulfide dibimane (SDB) and thiosulfate derivative pentafluorobenzyl (PFB)-S_2_O_3_H were determined [33,34]. Separation was performed in the Agilent Technologies 1290 HPLC system consisting of an autosampler and a binary pump. Chromatographic separation was performed on a Supelco C18 column (5 cm × 2.1 mm, 3 µm; Sigma–Aldrich, Bellefonte, PA, USA) protected by an Ascentis C18 column (2 cm × 2.1 mm, 3 µm; Merck KGaA, Darmstadt, Germany). The components were eluted by a gradient of A) 0.1% formic acid in water and B) acetonitrile. The flow rate was 300 μL/min. LC/MS was equipped with an electrospray ionization (ESI) source. Positive ionization mode for ESI source was used for detection. The data were collected in selected reaction monitoring mode using transitions of *m/z* 415—223, *m/z* 292.99—81, and *m/z* 212.99—93, for SDB, PFB-S_2_O_3_H, and PHB, respectively. The fecal concentrations of H_2_S and thiosulfate were represented in μg/g feces).

### 2.6. Metagenomics Analysis of Gut Microbiota

Bacterial DNA from frozen stool specimens was extracted and analyzed with metagenomics focused on the V3-V4 of the 16S DNA gene, as described previously [35]. The amplicons were sequenced on an Illumina MiSeq sequencer (Illumina, CA, USA) at the Genomic and Proteomic Core Laboratory, Kaohsiung Chang Gung Memorial Hospital (Kaohsiung, Taiwan). The sequences were analyzed while using QIIME version 1.9.1. Sequences with a distance-based similarity of 97% or greater were grouped into operational taxonomic units (OTUs) using the USEARCH algorithm. The phylogenetic relationships were determined based on a representative sequence alignment using Fast-Tree. We measured α-diversity as the observed richness and evenness of the taxa using the Shannon’s index [36,37]. We also quantified *β*-diversity as the variability in community composition across groups by the Principal Coordinate Analysis (PCoA). Additionally, the linear discriminant analysis effect size (LEfSe) was applied order to compare samples between groups and determine the significantly differential taxa. The threshold of the linear discriminant was set to 2.

### 2.7. Statistical Analysis

All of the data are expressed as mean ± the standard error of the mean (SEM). Statistical analysis of the data was performed using the Statistical Package for the Social Sciences software (SPSS Inc., Chicago, IL, USA). One-way analysis of variance and Tukey’s post hoc test was used for multiple comparisons. BP was analyzed by two-way repeated-measures analysis of variance and Tukey’s post hoc test. A probability value <0.05 was considered to be statistically significant. 

## 3. Results

### 3.1. Weights and Blood Pressures

**Figure 1:**
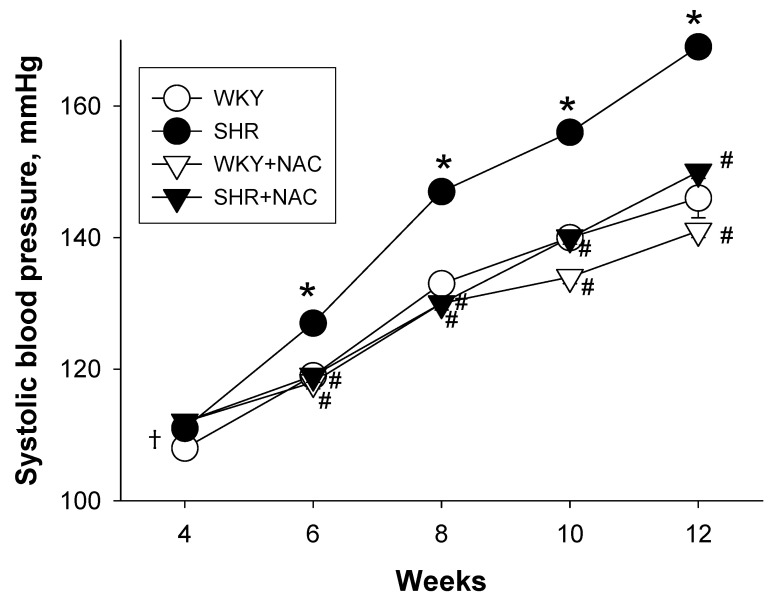
Effects of maternal N-acetylcysteine (NAC) therapy on systolic blood pressure. The data are shown as means ± SEM; N = 8/group. * *p* < 0.05 vs. WKY; ^#^ *p* < 0.05 vs. SHR. †In many instances the error bars of the figure are contained within the symbols.

No pups were dead in any of the groups, as shown in Table 2. The body weight (BW) of SHR was slightly greater than that of the WKY. Maternal NAC treatment caused an increase of BW in the WKY, but not SHR offspring. A higher kidney weight-to-BW ratio was found in the SHR and the SHR+NAC group compared to the WKY rats. Longitudinal measurement of systolic BP (SBP) showed that SHRs exhibited increased SBP as compared with the WKY rats from six to 12 weeks of age, as shown in Figure 1. The increases in SBP from six to 12 weeks of age were prevented by maternal NAC therapy. At 12 weeks of age, NAC also caused a slight reduction of SBP in the WKY rats (Table 1). The diastolic BP was comparable among the four groups. SHRs had a higher mean arterial pressure (MAP) vs. the WKY rats. Maternal NAC therapy resulted in a reduction of MAP as compared to the SHR at 12 weeks of age. 

### 3.2. Hydrogen Sulfide-Generating Pathway

**Figure 2:**
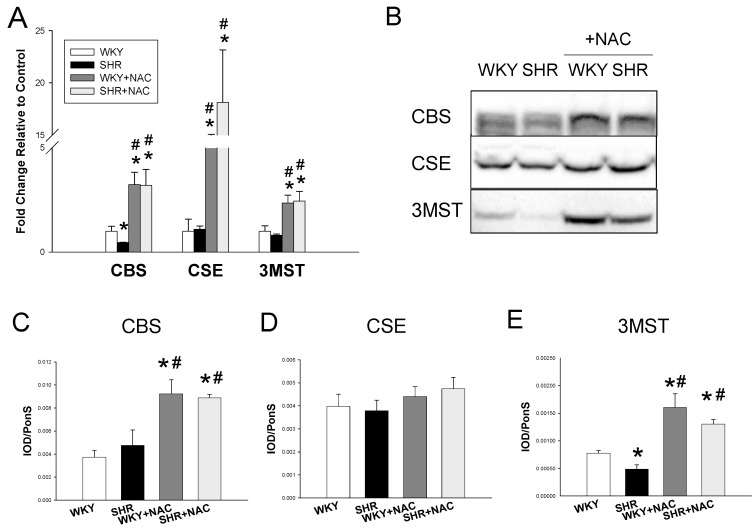
(**A**) Effect of maternal N-acetylcysteine (NAC) therapy on mRNA expression of H_2_S-generating enzymes in male offspring kidneys at 12 weeks of age. (**B**) Representative Western blots show cystathionine γ-lyase (CSE, ~45 kDa), cystathionine *β*-synthase (CBS, ~61 kDa), and 3-mercaptopyruvate sulfurtransferase (3MST, ~52 kDa) bands in male offspring kidneys at 12 weeks of age. Relative abundance of renal cortical (**C**) CSE, (**D**) CBS, and (**E**) 3MST were quantified. Data are shown as means ± SEM; N = 8/group. * *p* < 0.05 vs. WKY; ^#^ *p* < 0.05 vs. SHR.

**Figure 3:**
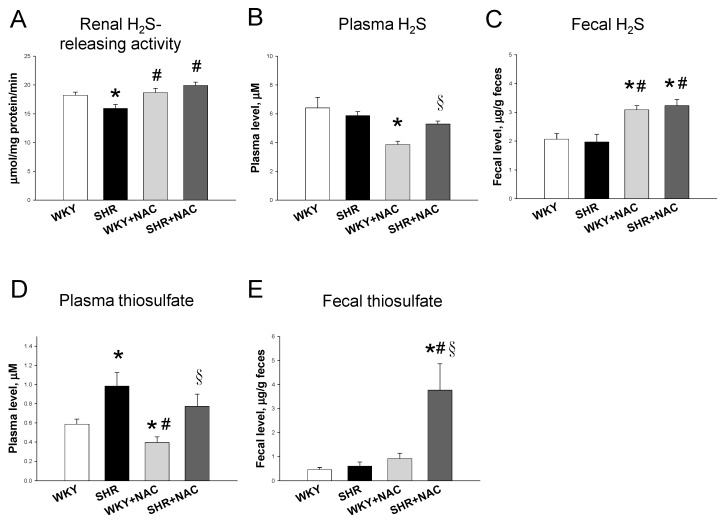
Effects of maternal N-acetylcysteine (NAC) therapy on (**A**) renal H_2_S-releasing activity, (**B**). Plasma H_2_S level, (**C**) fecal H_2_S level, (**D**) plasma thiosulfate level, and (**E**) fecal thiosulfate level. Data are shown as means ± SEM; N = 8/group. * *p* < 0.05 vs. WKY; ^#^ *p* < 0.05 vs. SHR; ^§^ *p* < 0.05 vs. WKY+NAC.

We first studied the renal mRNA expression of H_2_S-generating enzymes. SHRs had a lower CBS mRNA expression when compared to WKY rats, as shown in Figure 2A. Maternal NAC therapy significantly increased renal mRNA expression of CBS, CSE, and 3MST in WKY rats and SHRs treated with NAC as compared to those without treatment. We next analyzed protein levels of CBS, CSE, and 3MST in the kidney (Figure 2B). Maternal NAC treatment significantly increased renal CBS and 3MST protein levels in the WKY+NAC and SHR+NAC group (Figure 2C,E). We observed that renal CSE protein abundance was comparable among the four groups (Figure 2D). Additionally, renal H_2_S-releasing activity was lower in SHRs than that in WKY rats (Figure 3A). The producing rate of H_2_S in the kidney was higher in the WKY+NAC and SHR+NAC group as compared with that of WKY group. Furthermore, we determined the plasma and fecal concentrations of H_2_S and thiosulfate. The plasma H_2_S level was lowest in the WKY+NAC group among the four groups (Figure 3B). Maternal NAC therapy increased fecal H_2_S level in the WKY+NAC and SHR+NAC group compared to WKY and SHR group (Figure 3C). We observed that SHRs had a higher plasma thiosulfate level than WKY rats (Figure 3D). Likewise, the plasma thiosulfate level was higher in SHR+NAC group than that in WKY+NAC group. NAC therapy lowered plasma thiosulfate level in WKY rats, while it had a neglectable effect in SHRs. Moreover, the SHR+NAC group displayed the highest fecal thiosulfate level among the four groups. 

### 3.3. Renin-Angiotensin System

**Figure 4:**
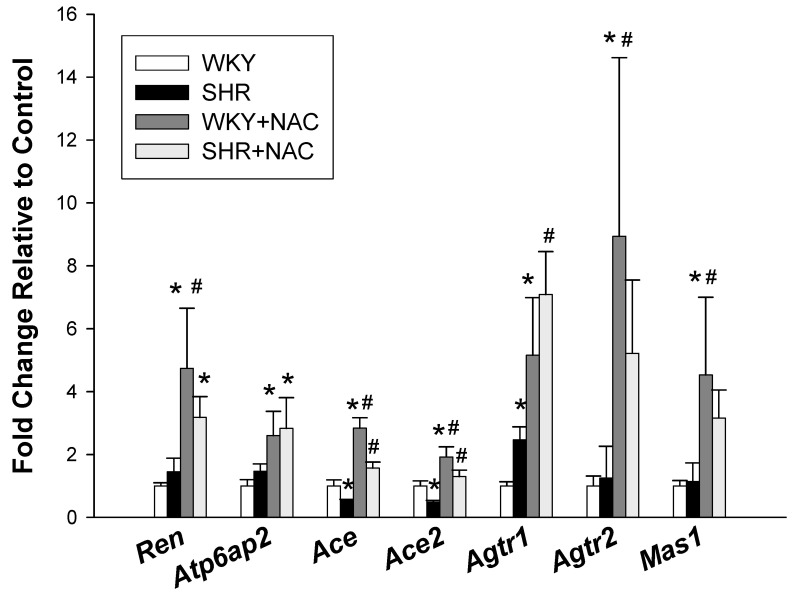
Effects of maternal N-acetylcysteine (NAC) therapy on the renin-angiotensin system. The data are shown as means ± SEM; N = 8/group. * *p* < 0.05 vs. WKY; ^#^ *p* < 0.05 vs. SHR.

We further evaluated the renal expression of the RAS components, including different angiotensin peptides that are mediated by their receptors (Figure 4). SHRs had a lower renal mRNA expression of *Ace* and *Ace2*, but a higher *Agtr1* mRNA expression compared with those in WKY rats. NAC treatment significantly increased renal mRNA expression of *Ren, Atp6ap2*, *Ace*, *Ace2*, *Agtr1*, *Agtr2*, and *Mas1* in WKY rats. In SHRs, NAC treatment caused increases of *Ace*, *Ace2*, and *Agtr1* expression in offspring kidneys. 

### 3.4. Gut Microbiota Compositions

**Figure 5:**
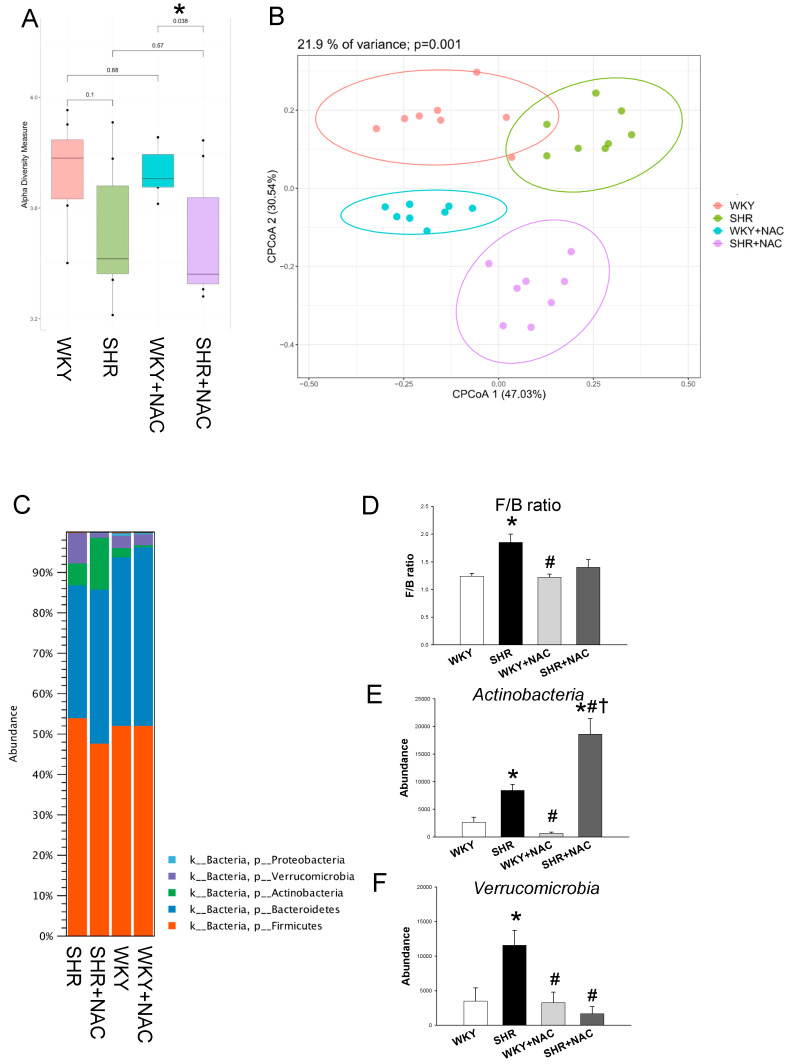
Effects of maternal N-acetylcysteine (NAC) therapy on the gut microbiome in male offspring at 12 weeks of age. (**A**) Variation in fecal bacterial α-diversity analyzed by the Shannon’s diversity indexes. (**B**) *β*-diversity changes in gut microbiota across groups by the Principal Coordinate Analysis (PCoA). (**C**) Relative abundance of the five major phyla of the gut microbiota among the four groups. In descending order, they are: *Firmicutes* (orange)*, Bacteroidetes* (blue)*, Actinobacteria* (green)*, Verrucomicrobia* (purple)*,* and *Proteobacteria* (light blue). (**D**) The *Firmicutes* to *Bacteroidetes* (F/B) ratio. Relative abundance of the phyla (**E**) *Actinobacteria* and (**F**) *Verrucomicrobia*. Abundance = the number of bacterial operational taxonomic unit (OTU) sequences detected in each sample. Data are shown as means ± SEM; N = 8/group. * *p* < 0.05 vs. WKY; ^#^ *p* < 0.05 vs. SHR; ^†^ *p* < 0.05 vs. WKY+NAC.

We further compared the difference in gut microbiota among the four groups at 12 weeks of age. Microbiome diversity is classically defined in terms of α-diversity (diversity within a community) and β-diversity (diversity between communities) [35]. The Shannon α-diversity index was not statistically different between the NAC treated and not treated WKY rats or SHRs, but there was a difference between the WKY+NAC and the SHR+NAC group (Figure 5A). We next performed *β*-diversity analysis techniques in order to compare the bacterial community similarity using the Principal Coordinate Analysis (PCoA). The scatterplots of PCoA analysis showed that all groups were well separated (Figure 5B, *p* = 0.001), indicating that four groups had distinct enterotypes. The major bacteria phyla found in 12-week-old offspring were *Firmicutes, Bacteroidetes, Actinobacteria, Verrucomicrobia,* and *Proteobacteria* (Figure 5C). The *Firmicutes* to *Bacteroidetes* (F/B) ratio has been considered to be a signature for hypertension [19]. Our data showed that the F/B ratio was higher in SHRs when compared with that in the WKY rats (Figure 5D). Maternal NAC therapy caused a remarkable increase in the phylum *Actinobacteria* compared to the other three groups (Figure 5E). Additionally, SHRs had a higher abundance of phylum *Verrucomicrobia*, which was restored by NAC treatment (Figure 5F). 

**Figure 6:**
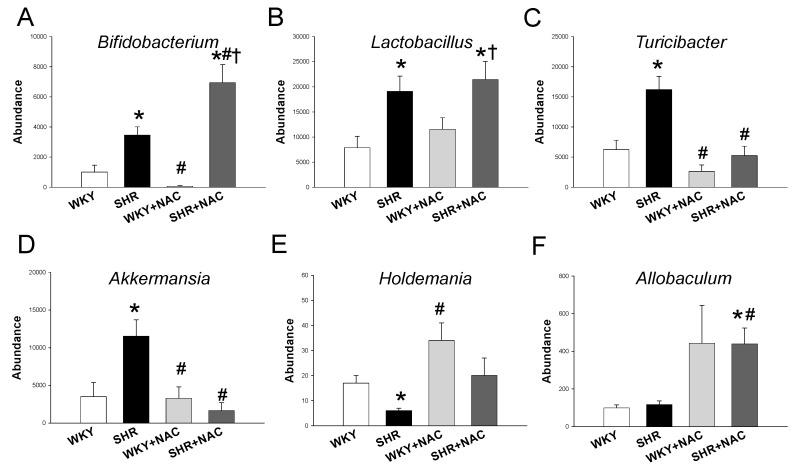
Effects of maternal N-acetylcysteine (NAC) therapy on the gut microbiome in male offspring at 12 weeks of age. Relative abundances of the genera (**A**) *Bifidobacterium*, (**B**) *Lactobacillus,* (**C**) *Turicibacter,* (**D**) *Akkermansia*, (**E**) *Holdemania*, and (**F**) *Allobaculum*. Abundance = the number of bacterial operational taxonomic unit (OTU) sequences detected in each sample. Data are shown as means ± SEM; N = 8/group. * *p* < 0.05 vs. WKY; ^#^ *p* < 0.05 vs. SHR; ^†^ *p* < 0.05 vs. WKY+NAC.

At the genus level, SHRs had a higher abundance of the genera *Bifidobacterium*, *Lactobacillus, Turicibacter,* and *Akkermansia* than in the WKY rats (Figure 6A,D). NAC therapy significantly increased the abundance of the genus *Bifidobacterium* (Figure 6A), while it decreased the abundance of genera *Turicibacter* (Figure 6C) and *Akkermansia* (Figure 6D) in the SHR+NAC group. Additionally, we observed that the genus *Holdemania* abundance was lower in SHRs when compared with that in the WKY rats. NAC therapy gave rise to a higher abundance of *Allobaculum* in the SHR+NAC group vs. SHR group. Moreover, the genera that belong to SRB (*Desulfovibrio, Desulfobacter, Desulfomonas, Desulfobulbus,* and *Desulfotomaculum*) were undetectable in either strain with or without NAC treatment.

**Figure 7:**
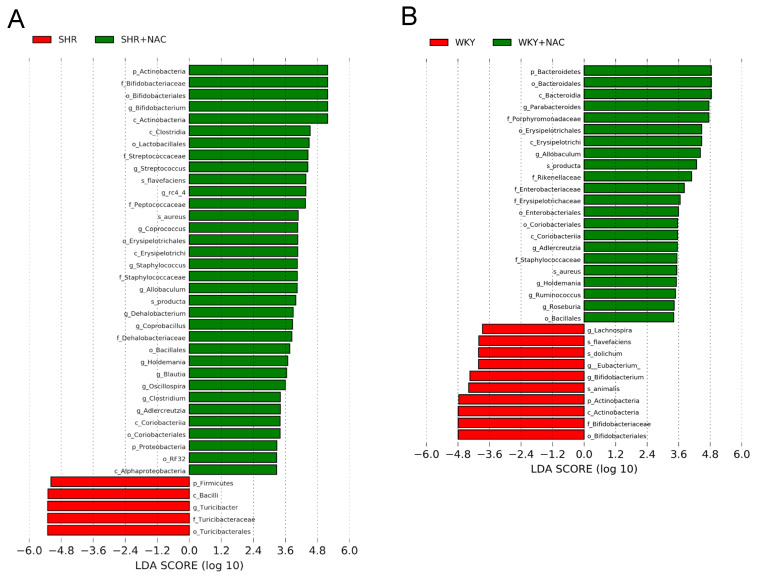
Effect of maternal N-acetylcysteine (NAC) therapy on the gut microbiome in male offspring at 12 weeks of age. Linear discriminant analysis effect size (LEfSe) was applied to identify enriched bacterial species. The threshold of the linear discriminant was set to 2. Different taxonomic levels of bacteria are given reaching from phylum (p) and class (c) via order (o) and family (f) down to genus (g) and species (s). Accordingly, the phylum Actinobacteria is presented as p_Actinobacteria (i.e., level_name). Most enriched and depleted bacterial taxa in the (**A**) SHR (red) versus SHR+NAC group (green) and (**B**) WKY (red) versus WKY+NAC group (green) are shown.

We further executed the linear discriminant analysis effect size (LEfSe) algorithm to the identified metagenomics biomarker (Figure 6). The LEfSe analysis identified a higher abundance of genera *Bifidobacterium*, *Allobaculum,* and *Holdemania*, but a lower abundance of genus *Turicibacter* in the SHR+NAC group vs. the SHR group (Figure 7A). Additionally, the WKY+NAC group had a higher abundance of *Allobaculum and Holdemania*, while a lower abundance of genus *Bifidobacterium* compared to the WKY group (Figure 7B).

## 4. Discussion

Our study provides new insights into the protective roles of maternal NAC therapy on hypertension through the regulation of the H_2_S-generating system and gut microbiota in SHRs. The major findings of our study can be summarized, as follows: (1) maternal NAC therapy prevented the elevation of SBP in male SHR offspring at 12 weeks of age; (2) maternal NAC therapy increased CBS and 3MST protein levels and H_2_S-releasing activity in SHR offspring kidneys; (3) beneficial effects of NAC were associated with marked increases of H_2_S and thiosulfate in feces; (4) NAC therapy enhanced several components in both classical and non-classical RAS axes in the WKY rats as well in SHRs; (5) maternal NAC therapy differentially shaped gut microbiota profile in SHRs and WKY rats. The four groups under consideration exhibited distinct enterotypes; (6) the protective effect of NAC against hypertension in SHRs is associated with increased phylum *Actinobacteria* and genera *Bifidobacterium* and *Allobaculum*, but decreased phylum *Verrucomicrobia*, genera *Turicibacter*, and *Akkermansia*; and, (7) the LEfSe analysis identified several microbial markers, including genera *Bifidobacterium*, *Allobaculum, Holdemania*, and *Turicibacter.*

In line with previous studies [14,16,38,39], our report supports the notion that H_2_S-generating system is involved in the development of hypertension in SHRs. In the current study, mother SHRs treated with NAC, the precursor of H_2_S, in pregnancy and lactation can increase renal H_2_S-generating activity and protein levels and prevent hypertension in their male adult offspring. The findings of this research are consistent with our previous study using a maternal suramin-induced programmed hypertension model, which indicated that maternal NAC therapy protects offspring against hypertension is related to increased renal 3MST protein level and H_2_S-releasing activity [20]. When comparing our results to those of previous studies using different maternal insults to program hypertension in adult offspring [19,20], it must be pointed out that the augmentation of renal H_2_S-generating pathway seems to be a common protective mechanism underlying maternal NAC therapy against the hypertension of developmental origins. Unlike previously reports that early NAC treatment prevented the transition from prehypertension to hypertension in young SHRs [21], our current report was the first to shift NAC therapy from early childhood to pregnancy to protect adult SHR offspring against the rise of BP. Our results go beyond previous reports [19,20,21], showing that the beneficial effects of NAC on hypertension were not only associated with renal H_2_S-generating system, but also marked increases of fecal H_2_S and thiosulfate levels in SHR offspring. 

Conflicting to previous reports showing H_2_S level was reduced in the SHRs [40,41], we did not find differences of fecal and plasma H_2_S level between SHRs and WKY rats. The diverse chemistries of H_2_S detection methods in the literature caused orders of magnitude differences in the measured physiological sulfide levels [24]. Most of the sulfide detected in previous studies was bound to different sulfide pools, leading to a relatively high concentration [38,40]. Our data collected in the HPLC-MS/MS analysis tie well with recently developed methods showing free sulfide levels are relatively low, as reported at low μM level in plasma [24]. We observed that the protective effect of NAC against hypertension is associated with increased fecal, but not plasma H_2_S level, in SHR offspring. Importantly, NAC significantly increased thiosulfate levels, an index of sulfide pool, in feces of SHRs. Our results suggest that increasing H_2_S bioavailability might be a potential approach to prevent hypertension and deserves further evaluation.

The beneficial effects of NAC on hypertension may also be attributed to shaping gut microbiota. We observed that the F/B ratio was only high in hypertensive SHRs, but not in other normotensive groups, which supports previous work in which this ratio was used as a microbial marker for hypertension [22]. In the current study, SHRs had higher abundance of lactate-producing genera *Lactobacillus* and *Turicibacter*, which was in line with previous reports showing that the lactate-producing bacteria population was increased in the SHRs [22]. The increased abundance of *Turicibacter* was restored by NAC therapy, while *Lactobacillus* was unaltered. The *Lactobacillus* species have been reported to be reduced in the presence of SRB-derived toxic H_2_S [41]. We found that nearly all SRB, including the principal SRB *Desulfovibrio,* were undetectable in either strain with NAC treatment. Conversely, NAC therapy significantly increased abundance of phylum *Actinobacteria*, one common SOB [24], and its related genus *Bifidobacterium*. Of note is that *Bifidobacterium*, a well-known beneficial microbe belonging to *Actinobacteria* phylum, was augmented by NAC therapy, which was in agreement with previous studies showing its benefit on hypertension [42,43]. Additionally, an antagonistic relationship between *Bifidobacterium* and SRB was reported in the sulfur metabolism [44]. Since that SOB can oxidize H_2_S to thiosulfate, and that NAC increased abundance of phylum *Actinobacteria* and fecal thiosulfate levels concurrently, our data suggest that the advantageous effects of NAC were due to, at least in part, increased SOB and their derived thiosulfate production. 

However, the data in this work demonstrated that other bacterial species that were involved in sulfur metabolism, such as *Streptococcus, Clostridium, Salmonella, Enterobacter*, *Fusobacterium,* and *Helicobacter,* were unaltered by NAC [45]. Interestingly, NAC therapy prevented the elevation of BP was associated with decreased genus *Akkermansia* abundance. Like *Lactobacillus*, *Akkermansia* is currently considered to be a beneficial microbe [42]. Our previous study reported that probiotic or prebiotic therapy that protected adult offspring against hypertension programmed by maternal high-fructose diet was related to increases of the abundance of *Akkermansia* [46]. Given that NAC increased fecal H_2_S levels, additional studies are required to clarify whether this discrepancy is a compensatory reduction of sulfur-metabolizing *Akkermansia* abundance in response to excessive colonic H_2_S. Although gut-derived H_2_S has been proposed for producing a systemic effect on BP [13], according to our data the protective effect of NAC on hypertension is mainly attributed to SOB and thiosulfate. SRB and other sulfur-metabolizing microbes only have a modest scale. 

Moreover, the beneficial effects of NAC therapy are related to the high abundance of genera *Holdemania* and *Allobaculum*. Our results tie well with previous studies showing that *Holdemania* and *Allobaculum* abundance was negatively correlated with SBP in SHR [47,48]. Nevertheless, there is still a need to validate above-mentioned microbial markers for hypertension identified in the current study in other hypertensive models.

Maternal NAC also mediated the RAS in both SHRs and WKY rats. The classic RAS, defined as the angiotensin converting enzyme (ACE)-Ang II-angiotensin type 1 receptor (AT1R) axis, promotes vasoconstriction and the rise of BP. Conversely, the non-classical RAS composed of the ACE2-Ang-(1-7)-Mas receptor axis is a counter-regulatory pathway in opposition to the vasoconstrictor arm of the RAS, leading to vasodilatation [32]. Thus, one might expect maternal NAC treatment to downregulate RAS components in classical RAS axis or upregulate non-classical RAS axis components, thereby mediating the RAS in a way that opposes the development of hypertension in adult offspring. In our hands, maternal NAC treatment of SHRs increased both *Ace2* and *Agtr1*. Whether the regulation of the RAS by NAC is a protective mechanism or a compensatory response in response to BP-lowering awaits further elucidation. 

One limitation in this study is that we have, so far, not checked whether the protective effect of NAC will extend beyond 12 weeks of age. Further studies addressing how long the effect of this protection last would be required. Secondly, we did not test other reported potential protective mechanisms of NAC on hypertension [18,30]. The antioxidant activity of NAC [49], such as reducing oxidative stress [21], improving NO bioavailability [19], and increased glutathione synthesis [50], against hypertension deserves further clarification. 

## 5. Conclusions

We conclude that maternal NAC therapy protects male adult offspring against hypertension, primarily through the regulation of the H_2_S-generating system and gut microbiota. Our data highlight the differential effects of maternal NAC therapy on H_2_S- generating system in the offspring kidneys and gut. The beneficial effects of NAC on hypertension are mainly associated with augmentation of renal H_2_S-generating system, increased SOB, and increased fecal thiosulfate levels in adult offspring. Although several microbial markers for hypertension were identified, their implications from our model awaits further validation in other models of developmental programming. The present study supports the assertion that maternal NAC therapy might prevent the hypertension of developmental origin.

## Figures and Tables

**Table 1:** List of primer sequences used for qPCR analysis in this study.

Gene	Forward (5′–3′)	Reverse (5′–3′)
*Cbs*	atgctgcagaaaggcttcat	gtggaaaccagtcggtgtct
*Cth*	cgcacaaattgtccacaaac	gctctgtccttctcaggcac
*Mpst*	ggctcagtaaacatcccattc	tgtccttcacagggtcttcc
*Ren*	aacattaccagggcaactttcact	acccccttcatggtgatctg
*Atp6ap2*	gaggcagtgaccctcaacat	ccctcctcacacaacaaggt
*Ace1*	caccggcaaggtctgctt	cttggcatagtttcgtgaggaa
*Ace2*	acccttcttacatcagccctactg	tgtccaaaacctaccccacatat
*Agtr1*	gctgggcaacgagtttgtct	cagtccttcagctggatcttca
*Agtr2*	caatctggctgtggctgactt	tgcacatcacaggtccaaaga
*Mas*	catctctcctctcggctttgtg	cctcatccggaagcaaagg
*Rn18S*	gccgcggtaattccagctcca	cccgcccgctcccaagatc

**Table 2:** Weights and blood pressures.

Group	WKY	SHR	WKY+NAC	SHR + NAC
Mortality	0%	0%	0%	0%
BW (g)	207 ± 5	230 ± 3 *	226 ± 4 *	217 ± 5
Left kidney weight (g)	0.90 ± 0.02	1.14 ± 0.02 *	1.07 ± 0.03 *	1.07 ± 0.02 *
Left kidney weight/100g BW	0.44 ± 0.01	0.5 ± 0.01 *	0.47 ± 0.01	0.49 ± 0.01 *
Systolic BP (mmHg)	146 ± 1	169 ± 1 *	141 ± 1 *^,#^	150 ±1 ^#^
Diastolic BP (mmHg)	76 ± 2	75 ± 3	72 ± 1	75 ± 2
MAP (mmHg)	99 ± 1	107 ± 2 *	95 ± 1 ^#^	100 ± 1 ^#^

Data are shown as means ± SEM; N = 8/group; BW = body weight. BP = blood pressure. MAP = mean arterial pressure. * *p* < 0.05 vs. WKY; ^#^ *p* < 0.05 vs. SHR.
